# P-1100. Activity of Cefiderocol and Comparator Agents Against Global Isolates of *Stenotrophomonas maltophilia* from the SENTRY Antimicrobial Surveillance Program (2020–2023)

**DOI:** 10.1093/ofid/ofae631.1288

**Published:** 2025-01-29

**Authors:** Motoyasu Onishi, Hidenori Yamashiro, Joshua Maher, Hank Kimbrough, Rodrigo E Mendes, Boudewijn L DeJonge, Sean T Nguyen, Christopher M Longshaw, Miki Takemura, Yoshinori Yamano

**Affiliations:** Shionogi & Co., Ltd., Toyonaka, Osaka, Japan; Shionogi & Co., Ltd., Toyonaka, Osaka, Japan; JMI Laboratories, North Liberty, Iowa; Element Materials Technology/Jones Microbiology Institute, North Liberty, Iowa; JMI Laboratories, North Liberty, Iowa; Shionogi Inc., Florham Park, New Jersey; Shionogi Inc., Florham Park, New Jersey; Shionogi B.V., London, England, United Kingdom; Shionogi & Co., Ltd, Toyonaka, Osaka, Japan; Shionogi & Co., Ltd., Toyonaka, Osaka, Japan

## Abstract

**Background:**

*Stenotrophomonas maltophilia* is a ubiquitous multidrug-resistant opportunistic pathogen. Infections caused by *S. maltophilia* have limited antibiotics treatment options due to the intrinsic resistant mechanisms such as the production of chromosomal L1 metallo-type carbapenemase and L2 extended-spectrum type β-lactamase. Cefiderocol (FDC) is a siderophore-conjugated cephalosporin with broad activity against Gram-negative bacteria, including *S. maltophilia*. In this study, the *in vitro* activities of cefiderocol and comparators were investigated against *S*. *maltophilia* clinical isolates that were collected from the US and Europe in 2020-2023 as part of the SENTRY Antimicrobial Surveillance Program.

Susceptibility of Stenotrophomonas maltophilia to cefiderocol and comparator agents

**Figure:**
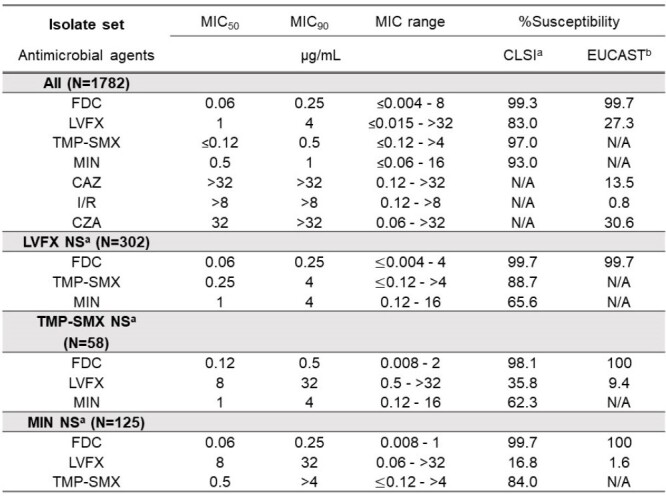

Cefiderocol (FDC), levofloxacin (LVFX), trimethoprim-sulfamethoxazole (TMP-SMX), minocycline (MIN), ceftazidime (CAZ), imipenem-relebactam (I/R), ceftazidime-avibactam (CZA), non-susceptible (NS), susceptible (S), intermediate (I), resistant (R), N/A (not applicable), a cefiderocol S ≤1, levofloxacin S ≤2, trimethoprim-sulfamethoxazole S ≤2/38, minocycline S ≤1, breakpoints as published by CLSI (2024), b cefiderocol S ≤2, levofloxacin S ≤0.5, ceftazidime S≤4, imipenem-relebactam S ≤2, ceftazidime-avibactam S ≤8, PK-PD breakpoints as published by EUCAST (2023)

**Methods:**

A total of 1782 *S. maltophilia* isolates were consecutively collected from the US and Europe from 2020-2023. The most common infection type from which isolates were collected was pneumonia (n=1193), followed by skin and skin structure (n=166), bloodstream infection (n=208), and urinary tract infections (n=56). Minimum inhibitory concentrations (MICs) of FDC and comparators were determined by using iron-depleted cation-adjusted Mueller-Hinton broth (ID-CAMHB) for cefiderocol and CAMHB for comparators. MIC_50_, MIC_90_, and MIC range were calculated. Susceptibility rates were determined by using CLSI breakpoints (2024) and EUCAST PK-PD breakpoints (2023).

**Results:**

FDC was the most potent agent tested against *S. maltophilia*, showing the lowest MIC_90_ value of 0.25 µg/mL (Table). The MIC_90_ values of levofloxacin (LVFX), trimethoprim-sulfamethoxazole (TMP-SMX), and minocycline (MIN) were 4, 0.5, and 1 µg/mL, respectively. The MIC_90_ values of other comparator agents were >8 µg/mL. Susceptibilities of FDC, LVFX, SMT, and MIN were 99.3, 83.0, 97.0, and 93,0%, respectively. The susceptibility to FDC was the highest against LVFX-, TMP-SMX- or MIN-non-susceptible isolates according to both CLSI and EUCAST breakpoint (Table).

**Conclusion:**

FDC showed potent *in vitro* activity against *S. maltophilia* collected in the US and Europe from 2020-2023, including isolates non-susceptible to LVFX, TMP-SMX, or MIN, suggesting that FDC is an important therapeutic option for *S. maltophilia* infections.

**Disclosures:**

**Motoyasu Onishi, PhD**, Shionogi & Co., Ltd.: Employee **Hidenori Yamashiro**, Shionogi & Co., Ltd.: Employee **Rodrigo E. Mendes, PhD**, GSK: Grant/Research Support **Boudewijn L. DeJonge, PhD**, Shionogi Inc.: Employee **Sean T. Nguyen, PharmD**, Shionogi Inc.: Employee **Christopher M. Longshaw, PhD**, Shionogi BV: Employee **Miki Takemura, n/a**, Shionogi & Co., Ltd.: Employee **Yoshinori Yamano, PhD**, Shionogi & Co., Ltd.: Employee

